# Anti-Inflammatory Effects of Spiramycin in LPS-Activated RAW 264.7 Macrophages

**DOI:** 10.3390/molecules27103202

**Published:** 2022-05-17

**Authors:** Jin-Kyu Kang, Hyun-Kyu Kang, Chang-Gu Hyun

**Affiliations:** Jeju Inside Agency and Cosmetic Science Center, Department of Chemistry and Cosmetics, Jeju National University, Jeju 63243, Korea; wlsrbtjsrb@naver.com (J.-K.K.); superxyz1993@naver.com (H.-K.K.)

**Keywords:** drug repurposing, inflammation, macrophages, mitogen-activated protein kinase (MAPK), nuclear factor κB (NF-κB), spiramycin

## Abstract

Drug repurposing is a simple concept with a long history, and is a paradigm shift that can significantly reduce the costs and accelerate the process of bringing a new small-molecule drug into clinical practice. We attempted to uncover a new application of spiramycin, an old medication that was classically prescribed for toxoplasmosis and various other soft-tissue infections; specifically, we initiated a study on the anti-inflammatory capacity of spiramycin. For this purpose, we used murine macrophage RAW 264.7 as a model for this experiment and investigated the anti-inflammatory effects of spiramycin by inhibiting the production of pro-inflammatory mediators and cytokines. In the present study, we demonstrated that spiramycin significantly decreased nitric oxide (NO), interleukin (IL)-1β, and IL-6 levels in lipopolysaccharide (LPS)-stimulated RAW 264.7 cells. Spiramycin also inhibited the expression of NO synthase (iNOS), potentially explaining the spiramycin-induced decrease in NO production. In addition, spiramycin inhibited the phosphorylation of mitogen-activated protein kinases (MAPKs); extracellular signal-regulated kinase (ERK) and c-Jun N terminal kinase (JNK) as well as the inactivation and subsequent nuclear translocation of nuclear factor κB (NF-κB). This indicated that spiramycin attenuates macrophages’ secretion of IL-6, IL-1β, and NO, inducing iNOS expression via the inhibition of the NF-κB and MAPK signaling pathways. Finally, we tested the potential application of spiramycin as a topical material by human skin primary irritation tests. It was performed on the normal skin (upper back) of 31 volunteers to determine whether 100 μM and μM of spiramycin had irritation or sensitization potential. In these assays, spiramycin did not induce any adverse reactions. In conclusion, our results demonstrate that spiramycin can effectively attenuate the activation of macrophages, suggesting that spiramycin could be a potential candidate for drug repositioning as a topical anti-inflammatory agent.

## 1. Introduction

Drug repurposing (or drug repositioning) can be defined as finding new therapeutic uses for older drugs that differ from their original medical indications. The discovery of novel molecular targets and pharmacological actions through drug repurposing strategies may provide new therapeutic opportunities for older drugs in clinical use [1]. The innovative advantage of a drug repurposing strategy is that it involves the basic knowledge of the existing drugs in terms of safety profiles, clinical uses, and manufacturing processes. In other words, it is possible to directly enter phase 2 clinical research, skipping preclinical development and phase 1 clinical trials [2]. This approach enables faster drug discovery by significantly reducing the investment and risk. In fact, bringing a small-molecule drug to the clinic is a long and expensive process, which is widely known to take 10 to 15 years on average, and can cost between USD 700 million and USD 2.7 billion. Advances in technology that can accelerate or significantly reduce costs will lead to a paradigm shift [3]. Successful examples of drug repositioning include the repurposing of Viagra, an erectile dysfunction drug consisting of sildenafil, designed as a hypertensive agent, and dimethyl fumarate, which has been prescribed for many years in Europe as a treatment for multiple sclerosis, into a psoriasis treatment, and remdesivir, the first COVID-19 treatment that received FDA approval in October 2020, which was undergoing the final clinical trials for treating Ebola virus infection [4]. The drug repurposing approach can be divided into experimental screening and in silico approaches [5]; we attempted to find a scientific clue for anti-inflammatories (new tricks) as new medicinal applications by targeting spiramycin (teaching an old dog), a classical antibiotic, through experimental screening in this study.

**Figure 1:**
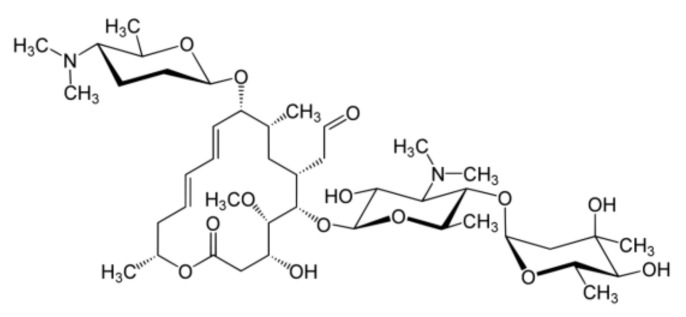
The structure of spiramycin.

Spiramycin is a 16-membered ring macrolide antibiotic that was first discovered 70 years ago in a culture medium of *Streptomyces ambofaciens* in 1952 (Figure 1). The antibiotic action of spiramycin, which inhibits protein synthesis in bacterial cells during translocation, has high antimicrobial activity against Gram-positive bacteria and mycoplasma species, and is widely used to treat toxoplasmosis and other soft-tissue infections in cattle, pigs, poultry, and sheep [6,7,8]. Spiramycin is prescribed in some countries for various odor-causing infections including periodontitis [9,10]. It has also been reported to exhibit antiviral activity against enterovirus A71 in vitro and in vivo, enabling its safe administration to children [11]. In regard to drug repurposing, several studies have reported that spiramycin effectively attenuates obesity and hepatic steatosis caused by a high-fat diet (HFD) by inhibiting adipogenesis [12]. The focus of this study was to investigate the inhibition of inflammatory mediators and cytokines including nitric oxide (NO), interleukin (IL)-6, and IL-1β, which is the anti-inflammatory capability of spiramycin in lipopolysaccharide (LPS)-induced RAW 264.7 macrophage cells. In addition, we demonstrated the possible intrinsic mechanisms, specifically through the mitogen-activated protein kinase (MAPK) and nuclear factor kappa-light-chain-enhancer of activated B cells (NF-κB), of the anti-inflammatory effect of spiramycin. These results suggest that through drug repurposing applications, spiramycin could be a candidate as an anti-inflammatory component in the future.

## 2. Results

### 2.1. Spiramycin Did Not Alter the Cell Viability in RAW 264.7 Cells

**Figure 2:**
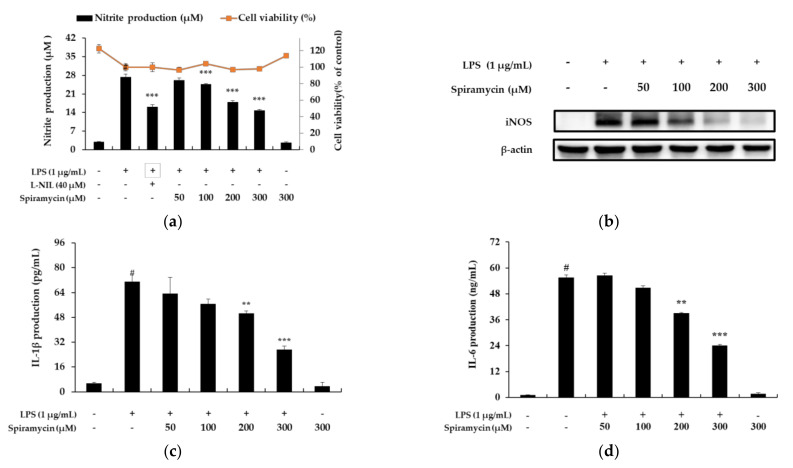
The effects of spiramycin on the inflammatory factor production and nitric oxide (NO) synthase (iNOS) expression in lipopolysaccharide (LPS)-stimulated RAW 264.7 cells. NO production (**a**) was determined by the Griess reagent method using L-N6-(1-Iminoethyl) lysine dihydrochloride (L-NIL) as a positive control. To measure the effect of spiramycin on the level of iNOS (**b**), lysates were prepared from cells pretreated with spiramycin (50, 100, 200, and 300 μM) for 1 h, and then treated with LPS (1 μg/mL) for 18 h. To assess the effect of spiramycin on interleukin (IL)-1β (**c**) and IL-6 (**d**) production, cells were pretreated with spiramycin for 1 h, and then stimulated with LPS for 20 h. The results are presented as the mean ± standard deviation (SD) from three independent experiments. Each experimental condition was performed in 10 parallel wells to ensure the reliability of the results. # *p* < 0.001 vs. the unstimulated control group. ** *p* < 0.01, *** *p* < 0.001 vs. LPS alone.

The cytotoxic effect of spiramycin on the RAW 264.7 cells was detected by the 3-(4,5-dimethylthizaol-2-yl)-2,5-diphenyl tetrazolium bromide (MTT) assay after treatment with LPS and various concentrations of spiramycin. The results showed that there were no differences among the 50, 100, 200, and 300 μM spiramycin-treated groups (Figure 2a), indicating that the cytotoxicity was not significantly affected by up to 300 μM spiramycin (>100% cell viability). Therefore, spiramycin at concentrations of 50, 100, 200, and 300 μM was used in the subsequent experiments.

### 2.2. Spiramycin Attenuates NO Production in RAW 264.7 Cells by the Inhibition of NO Synthase (iNOS) Expression

The amount of NO released when the RAW 264.7 macrophages were exposed to spiramycin was measured using the Griess reagent. As shown in Figure 2a, spiramycin treatment reduced NO production in a concentration-dependent manner. Next, to investigate whether the effect of spiramycin on NO production was mediated through the regulation of corresponding iNOS, we determined the protein expression level of iNOS by immunoblotting. As shown in Figure 2b, spiramycin treatment markedly downregulated the levels of iNOS protein expression in the RAW 264.7 cells in a concentration-dependent manner (50–300 μM). These results suggest that spiramycin was able to inhibit NO production by downregulating iNOS.

### 2.3. Spiramycin Attenuated Pro-Inflammatory Cytokine Production in the RAW 264.7 Macrophages

Cytokines are low-molecular-weight, water-soluble proteins, mainly produced through transcriptional and translational regulation through stimulation by immunogens, mitogens, or other promoters [13]. Here, the RAW 264.7 cells were treated with spiramycin for 24 h, and the protein levels of the cytokines were assessed using an enzyme-linked immunosorbent assay (ELISA) kit. As shown in Figure 2c,d, the addition of spiramycin resulted in marked decreases in IL-1β and IL-6 production in a concentration-dependent manner.

### 2.4. Spiramycin Attenuated the MAPK Signaling Pathway in the RAW 264.7 Cells

**Figure 3:**
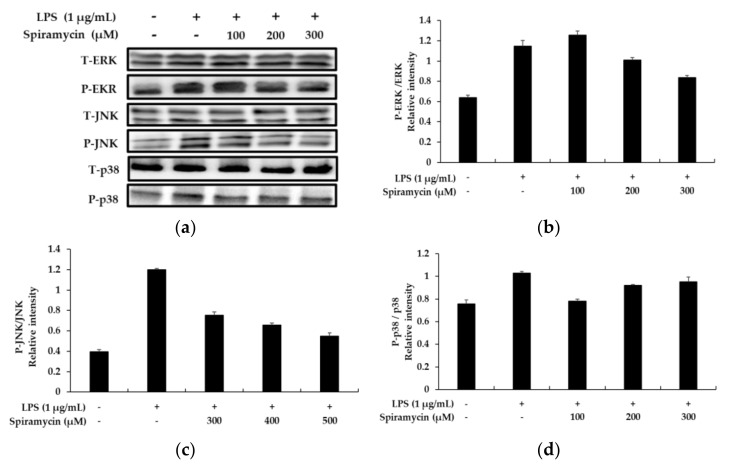
The effects of spiramycin on LPS-induced mitogen-activated protein kinase (MAPK) in the RAW 264.7 cells. Lysates were prepared from cells pretreated with spiramycin (100, 200, and 300 μM) for 1 h, and then treated with LPS (1 μg/mL) for 15 min. (**a**) Western blotting results, and protein expression of (**b**) P-ERK/T-ERK, (**c**) P-JNK/T-JNK and **(d**) P-p38/T-p38. β-actin was used as a loading control. The results are presented as the mean ± SD from three independent measurements using the Image J.

To further explore the mechanism of the anti-inflammatory effects of spiramycin, we investigated the effects of spiramycin on the MAPK signaling pathway by Western blotting. As shown in Figure 3, in the spiramycin-treated macrophage cells, the phosphorylation levels of extracellular-signal-related kinases (ERK1/2), and c-Jun N-terminal kinases (JNKs) were significantly attenuated in a concentration-dependent manner, with downregulated JNK being the most pronounced. However, spiramycin only slightly reduced the LPS-induced p38 phosphorylation.

### 2.5. Spiramycin Attenuated the NF-κB Signaling Pathway in the RAW 264.7 Cells

**Figure 4:**
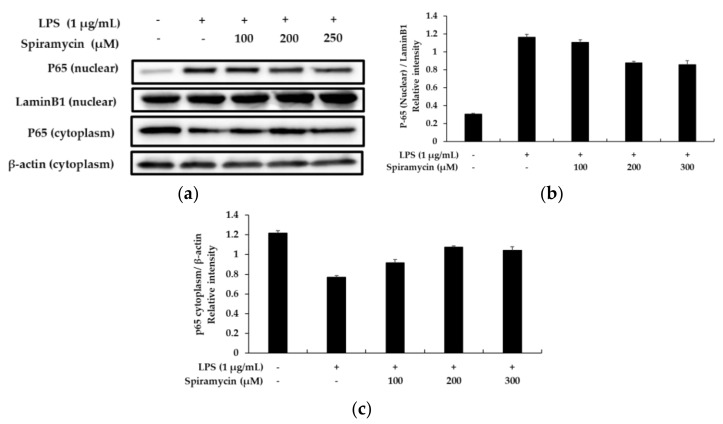
The effect of spiramycin on the level of nuclear factor κB (NF-κB; p-65; nucleus) and (p-65; cytoplasm) in LPS-induced RAW 264.7 cells. Lysates were prepared from cells pretreated with spiramycin (100, 200, and 300 μM) for 1 h, and then treated with LPS (1 μg/mL) for 15 min. (**a**) Western blotting results, and protein expression of (**b**) p65 (nuclear) and (**c**) p65 (cytoplasm). β-actin and Lamin B1 were used as a loading control. The results are presented as the mean ± SD from three independent measurements using the Image J.

**Figure 5:**
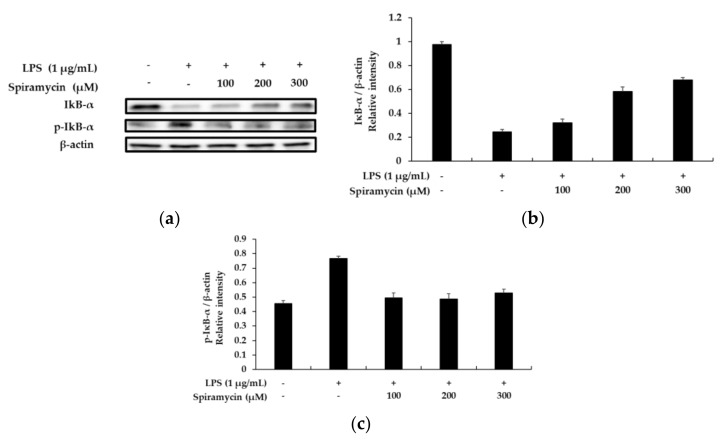
The effect of spiramycin on the levels of phospho-IκB-α and IκB-α in the LPS-induced RAW 264.7 cells. Lysates were prepared from cells pretreated with spiramycin (100, 200, and 300 μM) for 1 h, and then treated with LPS (1 μg/mL) for 20 min. (**a**) Western blotting results, and protein expression of (**b**) IκBα and (**c**) P-IκBα. β-actin was used as a loading control. The results are presented as the mean ± SD from three independent measurements using the Image J. β-actin was used as a loading control.

NF-κB is a major regulator and a major transcription factor regulating the expression of various cytokines; therefore, investigations were performed to determine whether spiramycin treatment could inhibit the nuclear translocation of the NF-κB p65 subunit in the RAW 264.7 macrophages. As depicted in Figure 4, the nuclear level of the p65 protein decreased in the spiramycin-treated cells, whereas the cytoplasmic level increased in a concentration-dependent manner. In addition, in many mechanistic studies related to the NF-κB pathway, it has been reported that LPS treatment induces the degradation of IκB-α to activate the NF-κB pathway. In this regard, as shown in Figure 5, our data showed that the degradation of IκB-α by LPS could be significantly reversed by spiramycin treatment. Together, our findings suggest that the anti-inflammatory effect of spiramycin is associated with the inhibition of the NF-κB pathway.

### 2.6. Spiramycin Did Not Induce Any Adverse Reactions in Human Skin Primary Irritation Test

To evaluate the irritation effects of spiramycin (100 μM and 300 μM) on human skin, patch tests were performed. As shown in Table 1, we did not observe any severe adverse reactions such as slight erythema, burning, or pruritus related to topical treatment with spiramycin.

## 3. Discussion

For decades, drug discovery has been driven by a one drug/one target approach, also known as a ‘lock-and-key’ model, which excludes all ‘off-targets’ that can have the dominant principle of drug design. However, this principle has recently shifted from the ‘one drug/one target’ model to the ‘one drug/multi-target’ model, which is known as ‘multi-pharmacology’ [14]. On one hand, drug repurposing has the potential to cause side effects due to off-target interactions with drugs. Nonetheless, it is undeniable that drug repurposing also creates opportunities to discover new treatments. Most of the success stories of drug repurposing such as with Viagra have occurred through accidental discoveries that deviated from the ‘one-drug/one-target’ model [4]. However, the same success can now be achieved through a more deliberate and systematic approach.

In this study, we confirmed the possibility of applying an old weapon, spiramycin, to various inflammatory diseases, the new enemy. Spiramycin has been shown to exhibit anti-bacterial, anti-viral, and anti-parasitic activities [6,7,8,9,10,11,12]. However, no study has reported the anti-inflammatory potential of spiramycin in LPS-induced macrophage RAW 264.7 cells. Inflammation models through LPS exposure to macrophages have been used extensively for the discovery and study of the underlying mechanisms of anti-inflammatory drugs. In this study, we also found that spiramycin treatment inhibited LPS-induced macrophage activation using this model as follows. First, we found that spiramycin inhibited NO production by suppressing iNOS protein expression. NO, a pro-inflammatory mediator biosynthesized by the iNOS protein, is secreted by macrophage cells, and first triggers the inflammatory response, increases vascular permeability, and stimulates macrophages to produce various pro-inflammatory cytokines and mediators [15]. Thus, the influences of spiramycin on iNOS proteins were measured to investigate its anti-inflammatory properties; spiramycin exhibited significant inhibitory effects on the LPS-induced upregulation of iNOS expression. These results suggest that spiramycin potently inhibits the expression of pro-inflammatory mediators, thereby inhibiting the initiation of inflammatory disease or improving exacerbated inflammation.

Second, we found that treatment with spiramycin reduced the secretion of IL-1β and IL-6. Several pro-inflammatory cytokines such as tumor necrosis factor (TNF-α), IL-6, or IL-1β play an important role in the innate immune response. These pro-inflammatory cytokines produced by macrophages during inflammation are important factors in the development and progression of chronic inflammation [16]. Our results showed that spiramycin reduced IL-6 and IL-1β production in the LPS-stimulated mouse macrophage cells. Third, spiramycin displayed a strong inhibitory effect against the LPS-induced activation of the MAPK/NF-κB signaling pathway. Nuclear factor κB and MAPKs are two representative intracellular signaling pathways that can regulate the expression of many inflammatory mediators and cytokines [15,16,17,18]. Therefore, we analyzed proteins related to the NF-κB and MAPK pathways to investigate the anti-inflammatory effects of spiramycin and its association with the above-mentioned signaling pathways. LPS challenge induces the activation of IκB-α phosphorylation, an inhibitor of NF-κB; subsequently, the nuclear translocation of p65, a subunit of NF-κB, is induced, thereby activating the transcription of target genes such as iNOS. Moreover, increasing evidence indicates that LPS triggers the degradation of IκB-α, thereby activating the NF-κB pathway. Our data showed that the expression level of the IκB-α protein was significantly reduced in cells treated with LPS, which was remarkably reversed by treatment with spiramycin. In addition, the nuclear level of the p65 protein was decreased in the spiramycin-treated cells; thus, the cytoplasmic level of the p65 protein increased in a concentration-dependent manner, indicating that the anti-inflammatory effect of spiramycin is related to the inhibition of NF-κB. Activation of the MAPK pathway plays an essential role in the initiation and development of inflammatory diseases that are mediated by sequential phosphorylation events [17,18]. Our results showed that spiramycin markedly decreased the LPS-stimulated phosphorylation of the ERK and JNK proteins, which are involved in regulating the expression of pro-inflammatory mediators and cytokine genes. These results indicate that ERK and JNK, elements of the MAPKS signaling pathway, are involved in the anti-inflammatory effect of spiramycin. In summary, spiramycin suppresses the expression of inflammatory mediators such as iNOS as well as decreases the production of the inflammatory cytokines NO, IL-1β, and IL-6 through the downregulation of the NF-κB and MAPK signaling pathways in LPS-induced macrophages. These results suggest that spiramycin, a classic antibiotic, may be a potential treatment for various inflammatory diseases.

From a drug repurposing perspective, the anti-inflammatory effect of spiramycin may provide several important benefits such as, first and foremost, its applicability in the treatment of coronavirus disease 2019 (COVID-19). The emergence of COVID-19-mediated cytokine storms that cause the secretion of a large number of proinflammatory factors such as IL-6, IL-1β, TNF-α, and chemokines, which are considered important factors leading to death in COVID-19 patients, is one of the most important contributors to the incidence of acute and severe diseases in patients [19]. Currently, drugs capable of inhibiting cytokine storms are urgently needed to treat COVID-19. Zhen et al. reported that spiramycin significantly reduced the enterovirus A71 (EV-A71) and coxsackievirus A16 (CV-A16) RNA and protein levels, most likely by interfering with viral RNA replication. As a potential treatment option for EV-A71- and/or CV-A16-induced hand, foot, and mouth disease, it has been claimed to be safe in infants and children [11]. Our results showed that spiramycin has strong inhibitory effects on IL-6, IL-1β, NO, and iNOS as well as an antiviral treatment, suggesting the possibility of spiramycin as a treatment for cytokine storms. Second, we investigated the applicability of spiramycin as a treatment for skin disorders. Skin appearance is essential for self-esteem and quality of life. As a result, skincare products represent a large market [20]. Acne vulgaris, vaginitis, seborrheic dermatitis, and dermatophytosis, which are representative skin problems, are the main causes of skin pathogens and the accompanying chronic inflammation. Therefore, if the anti-inflammatory spiramycin is equipped with a new anti-inflammatory effect, it can hold potential as a new treatment for skin diseases. Third is the potential of spiramycin as an anti-obesity treatment and cosmeceutical ingredient. Kim et al. reported that HFD-induced obese mice administered with spiramycin showed significant reductions in weight gain, serum leptin levels, adipose tissue mass, and liver lipid accumulation, which effectively attenuated high-fat diet (HFD)-induced obesity and hepatic steatosis by inhibiting adipogenesis [12]. In addition, according to our unreported results, spiramycin has potential as a whitening cosmeceutical ingredient by inhibiting melanin production in B16F10 melanoma cells.

In summary, in terms of drug repurposing, our research target, spiramycin, is an interesting drug as an anti-inflammatory, anti-obesity, and cosmeceutical ingredient, in addition to antibiotic and antiviral treatments. Moreover, spiramycin did not induce any severe adverse reactions in the human skin irritation tests. Considering these results, we suggest that spiramycin could be considered as a possible anti-inflammatory candidate for topical application. Further research is required to determine its anti-inflammatory properties against skin diseases such as acne and atopic dermatitis.

## 4. Materials and Methods

### 4.1. Chemicals and Reagents

Spiramycin, LPS from *Escherichia coli*, MTT, dimethyl sulfoxide (DMSO), Griess reagent, sodium nitrite, protease inhibitor cocktail, and phosphate-buffered saline (PBS) were obtained from Sigma-Aldrich (St. Louis, MO, USA). Cytoplasmic extraction reagents (NCER), Dulbecco’s modified Eagle’s medium (DMEM), antibiotics (penicillin/streptomycin solution), and fetal bovine serum (FBS) were obtained from Thermo Fisher Scientific (Waltham, MA, USA). Enzyme-linked immunosorbent assay kits for prostaglandin E_2_ (PGE_2_), IL-1β, interleukin-6, and TNF-α were purchased from R&D Systems Inc. (Minneapolis, MN, USA). Antibodies against β-actin, anti-iNOS, anti-COX-2, p65 (Ser536), p-p65 (Ser32), and anti-NF-κB (IκBα) were purchased from Calbiochem (San Diego, CA, USA). T-ERK, P-ERK, T-JNK, P-JNK, T-P38, and P-P38 were obtained from Cell Signaling Technology (Beverly, MA, USA). All other reagents were of analytical grade.

### 4.2. Cell Culture

The RAW 264.7 murine macrophage cells were purchased from the Global BioResource Center (Carlsbad, CA, USA). The RAW 264.7 murine macrophage cells were sub-cultured at intervals of 2 d using DMEM with 10% FBS and 1% antibiotics (penicillin/streptomycin) at 37 °C in a 5% CO_2_ incubator (N-BIOTEK, NB-203XL).

### 4.3. Cell Viability

Cell viability was measured using the MTT assay. Cells (1.5 × 10^5^ cells/well) were seeded in a 24-well plate and incubated for 24 h. The cells were then treated with various concentrations of spiramycin (50, 100, 200, and 300 μM) for 24 h. Next, the MTT reagent was treated at a concentration of 0.4 mg/mL in 24-wells, and the medium was removed after incubation for 2 h. Finally, 1 mL of DMSO was added, and absorbance was measured at 570 nm using a microplate reader (Tecan, Mannedorf, Switzerland).

### 4.4. NO Production

Cells (1.5 × 10^5^ cells/well) were seeded in a 24-well plate and incubated for 24 h. The cells were pretreated with different concentrations of spiramycin (50, 100, 200, and 300 μM) for 1 h, and then treated with LPS (1 μg/mL) for 24 h. Thereafter, the cell supernatant and Griess reagent (1% sulfanilamide, 0.1% N-1-(naphthyl) ethylenediamine dihydrochloride, 2.5% phosphoric acid) were mixed in a 1:1 ratio and incubated for 20 min. Absorbance was measured at 540 nm using a microplate reader (Tecan, Mannedorf, Switzerland). The amount of NO produced was quantitatively calculated using the standard curve of sodium nitrite (NaNO_2_).

### 4.5. Measurement of Cytokines

Cells (1.5 × 10^5^ cells/well) were inoculated in a 24-well plate and incubated for 24 h. The cells were pretreated with various concentrations of spiramycin (50, 100, 200, and 300 μM) for 1 h, and then treated with LPS (1 μg/mL) for 24 h. The supernatants were collected, and the levels of IL-1β and IL-6 were measured using ELISA systems, according to the manufacturer’s protocol.

### 4.6. Western Blot Analysis

The RAW 264.7 murine macrophage cells were plated at a density of 6.0 × 10^5^ cells/dish in 60 mm cell-culture dishes for 24 h. The cells were pretreated with various concentrations of spiramycin (50, 100, 200, and 300 μM) for 1 h and treated with LPS (1 μg/mL) for the specified time. After incubation, the cells were washed twice with cold 1X PBS. Next, RIPA lysis buffer was added and stored at low temperature for 20 min. The lysate was transferred to a microtube and centrifuged at −4 °C, 13,000 rpm, for 15 min. The lysate was transferred to an Eppendorf tube and centrifuged at −4 °C, 12,000 rpm, for 15 min. The protein content of the supernatant was quantitatively calculated using a standard calibration curve for bovine serum albumin (BSA) using a bicinchoninic acid protein assay kit, which measured the absorbance at 562 nm to assess the degree of purple color development by the oxidized copper bond. Whole-cell protein extracts (25 μg) were separated by sodium dodecyl sulfate-polyacrylamide gel electrophoresis (SDS-PAGE) on a 10% gel, and transferred onto a PVDF membrane by applying an electric field. Membranes were blocked for 1 h at room temperature in 1X TBST with 5% non-fat skim milk, washed six times at 10 min intervals with TBST, a mixture of tris-buffered saline (TBS) buffer and 0.1% Tween 20, and incubated for 8 h at 4 °C with the appropriate primary antibody (1:2000). Thereafter, the membrane was washed six times every 10 min with TBST, and an appropriate secondary antibody was incubated at room temperature for 2 h. Finally, the membrane was washed six times with TBST buffer every 10 min, and the proteins were detected using an enhanced chemiluminescence (ECL) kit.

### 4.7. Preparation of Nuclear and Cytosolic Extraction

The RAW 264.7 murine macrophage cells were inoculated at a density of 6.0 × 10^5^ cells/dish in 60 mm cell-culture dishes for 47 h. The cells were pretreated with various concentrations of spiramycin (50, 100, 200, and 300 μM) for 1 h and treated with LPS (1 μg/mL) for 15 min. Next, measurements were performed using nuclear and cytoplasmic extraction reagents (NE-PER^TM^), according to the manufacturer’s protocol.

### 4.8. Human Skin Primary Irritation Test

The human skin primary irritation test was commissioned by Dermapro Co. Ltd. (Seoul, Korea) and performed as follows. Thirty-one healthy female Korean subjects were selected based on the inclusion and exclusion criteria, and written consent was obtained in each case. The average age was 43.2 years (range: 29–53). The subjects had no history of allergic contact dermatitis, nor had they used topical or systemic irritant preparations in the previous 2 weeks. The spiramycin formulated with squalene were prepared and applied at 100 μM and 300 μM concentrations. After washing the upper back of the subjects with 70% ethanol, 20 μL of spiramycin was applied to the subjects for 24 h. The first evaluation was conducted 20 min after the patch removal, and the second evaluation was conducted 24 h later. The skin primary irritation reactivity was evaluated according to Personal Care Products Council (PCPC) guidelines (2014).

### 4.9. Statistical

All experimental results were expressed as the mean ± SD from three independent experiments. Statistical analysis was performed using the Student’s *t*-test, in Microsoft Office Excel or GraphPad Prism software. *p* < 0.05 was considered as statistically significant.

## Figures and Tables

**Table 1:** The evaluation of spiramycin for primary skin irritation potential in humans.

Test Sample	No. of Responder	20 min after Patch Removal				24 h after Patch Removal				Reaction Grade (R) Mean
		+1	+2	+3	+4	+1	+2	+3	+4	0.0
Spiramycin 100 μM	0	-	-	-	-	-	-	-	-	0.0
Spiramycin 300 μM	0	-	-	-	-	-	-	-	-	0.0
Squalene	0	-	-	-	-	-	-	-	-	0.0

## Data Availability

Not applicable.

## Sample Availability

Not available.
